# Supplementary material for: Human Anelloviruses: Influence of Demographic Factors, Recombination, and Worldwide Diversity
Source: Microbiol Spectr. 2023 May 18;11(3):e04928-22. doi: 10.1128/spectrum.04928-22 (PMC10269794; doi:10.1128/spectrum.04928-22)

Supplementary Figure S1. Global phylogenetic tree for the ORF1 of the three anellovirus genera. Downloaded sequences defined as reference species and sequences described in this study (labelled in red) are included. 0.7-0.85 and 0.85-1.0 bootstrap value ranges are indicated with blue and red circles, respectively. The scale bar indicates the evolutionary distance in nucleotide substitutions per site.

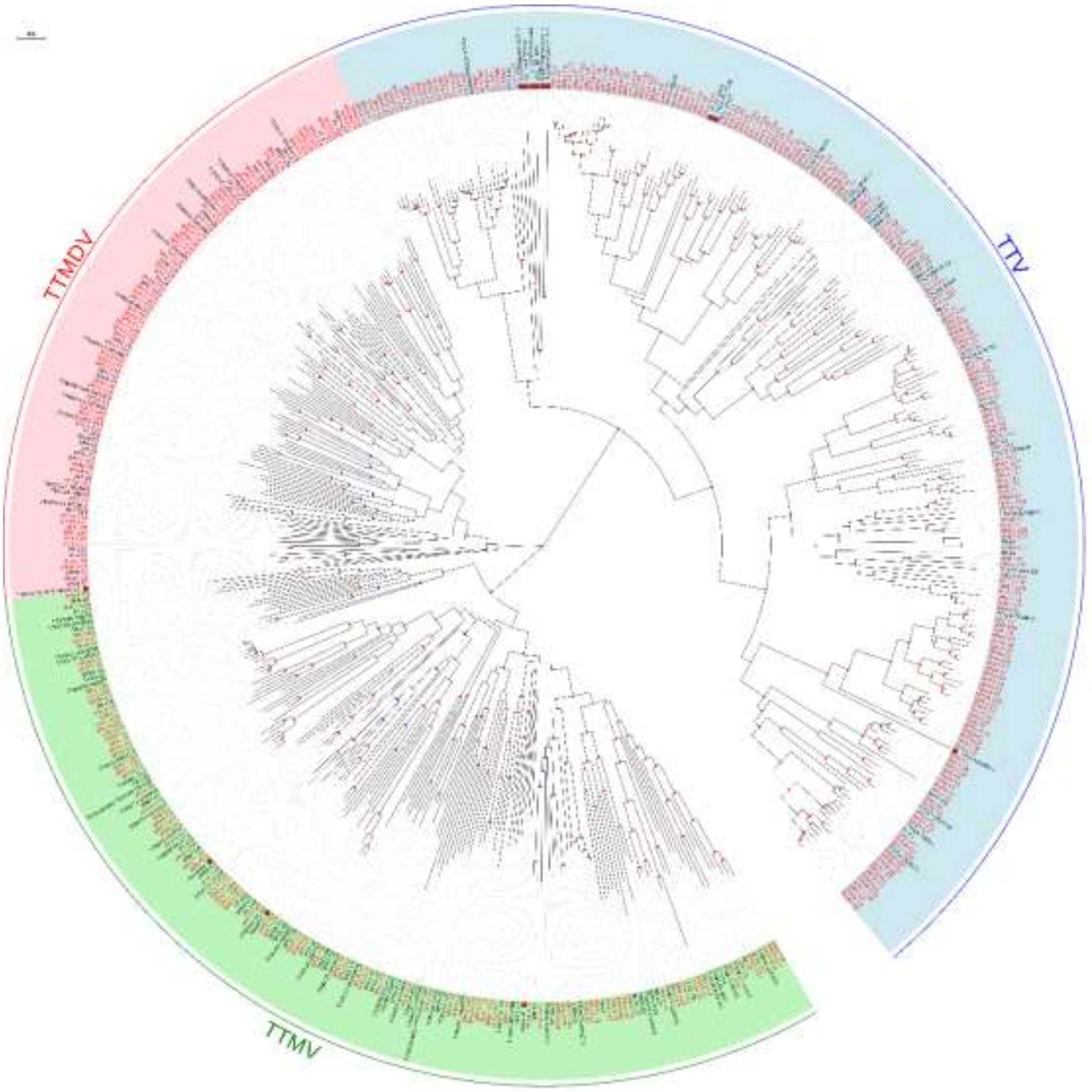

Supplement: Supplemental file 6 — Fig. S1. Download spectrum.04928-22-s0006.pdf, PDF file, 0.2 MB [file spectrum.04928-22-s0006.pdf]
